# Comparative proteomic analysis of normal and tumor stromal cells by tissue on chip based mass spectrometry (toc-MS)

**DOI:** 10.1186/1746-1596-5-10

**Published:** 2010-01-28

**Authors:** Niko Escher, Günther Ernst, Christian Melle, Alexander Berndt, Joachim H Clement, Kerstin Junker, Karlheinz Friedrich, Orlando Guntinas-Lichius, Ferdinand von Eggeling

**Affiliations:** 1 Core Unit Chip Application, Institute of Human Genetics, University Hospital Jena, 07740 Jena, Germany; 2 Institute of Pathology, University Hospital Jena, 07740 Jena, Germany; 3 Department of Hematology and Oncology, Clinic for Internal Medicine II, University Hospital Jena, 07740 Jena, Germany; 4 Department of Urology, University Hospital Jena, Lessingstrasse 1, 07740 Jena, Germany; 5 Institute of Biochemistry II, University Hospital Jena, Nonnenplan 2, 07740 Jena, Germany; 6 Department of Otorhinolaryngology, University Hospital Jena, 07740 Jena, Germany

## Abstract

In carcinoma tissues, genetic and metabolic changes not only occur at the tumor cell level, but also in the surrounding stroma. This carcinoma-reactive stromal tissue is heterogeneous and consists e.g. of non-epithelial cells such as fibroblasts or fibrocytes, inflammatory cells and vasculature-related cells, which promote carcinoma growth and progression of carcinomas. Nevertheless, there is just little knowledge about the proteomic changes from normal connective tissue to tumor stroma. In the present study, we acquired and analysed specific protein patterns of small stromal sections surrounding head and neck cell complexes in comparison to normal subepithelial connective tissue. To gain defined stromal areas we used laser-based tissue microdissection. Because these stromal areas are limited in size we established the highly sensitive 'tissue on chip based mass spectrometry' (toc-MS). Therefore, the dissected areas were directly transferred to chromatographic arrays and the proteomic profiles were subsequently analysed with mass spectrometry. At least 100 cells were needed for an adequate spectrum. The locating of differentially expressed proteins enables a precise separation of normal and tumor stroma. The newly described toc-MS technology allows an initial insight into proteomic differences between small numbers of exactly defined cells from normal and tumor stroma.

## Findings

Carcinoma tissue does not only consist of tumor cells but also of fibroblasts, endothelial cells or vascular structures, and inflammatory cells forming the so-called desmoplastic stroma reaction or supportive tumor stroma. Many steps in carcinoma development e.g. proliferation, angiogenesis, invasion and metastasis are promoted by microenvironmental factors produced by these stromal cells. It is well known that the reciprocal interactions between tumor and stroma cells, i.e., cancer associated fibroblasts (CAF), tumor endothelial cells (TEC) and tumor associated macrophages (TAM) result in tumor progression. The close vicinity of CAFs to the cancer cells enhance tumor growth by secreting growth factors like transforming growth factor beta (TGF beta), matrix degrading enzymes like matrix metalloproteinases (MMP) and angiogenic factors such as vascular endothelial growth factors (VEGF) [1]. The investigation of those microenvironmental factors at the proteomic level requires a technical workflow that enables the isolation of small defined areas of stroma on the one hand and a sufficient high sensitivity to analyse these small amounts of cells on the other hand. One part of this attempt is the laser-based tissue microdissection [2]. Hereby, small areas of interest can be easily separated from the remaining tissue and further analyzed with genomic or proteomic approaches. The second prerequisite for the proteomic analysis of stromal cells is a highly sensitive detection technique. Gel-based techniques do not meet this requirement but mass spectrometry by MALDI (matrix assisted laser desorption and ionization) seems to be a better choice as shown in several studies using microdissected tissue [2,3]. Using affinity chromatographic surfaces SELDI (surface enhanced laser desorption and ionization) offers the highest sensitivity - but with low resolution - and is a commonly used tool to investigate differentially expressed proteins in body fluids, cells and tissue [4-9]. In general, SELDI is useful to compare crude protein lysates with a high sensitivity; MALDI, in contrast, displays a higher resolution which is useful for the identification of proteins. So far, after microdissection about 3000-5000 cells are needed to receive an adequate proteomic profile. Nevertheless, it is tedious to reach even this cell number from small stromal areas within a tumor. Therefore, the purpose of this study was to develop and refine a proteomic technique which is sensitive enough to analyse as few as a hundred microdissected cells.

### Microdissection of stroma from normal and tumor tissue

All head and neck tumor samples (n = 14) and normal controls (n = 14) were obtained after surgical resection at the ENT (Ear, Nose, Throat) Department of the University Hospital Jena; they had been collected fresh, snap frozen in liquid nitrogen, and were stored at -80°C. Tumor specimen were categorized to the WHO classification criteria [10]. Ethical approval was obtained from the local Research Ethic Committee.

**Figure 1:**
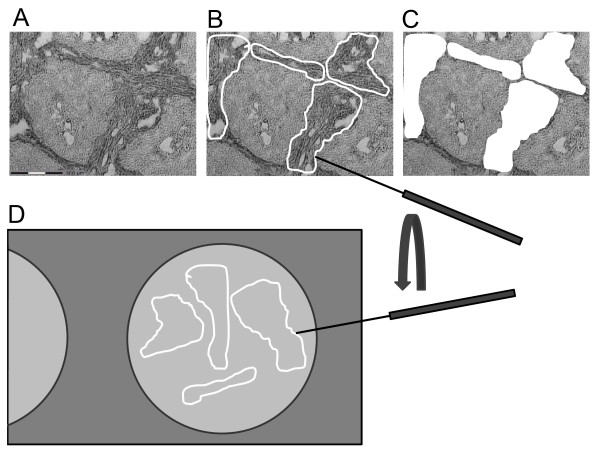
**Principle of tissue on chip based mass spectrometry (toc-MS): (A) Head and neck cancer (HNC) tissue sections were stained H&E to obtain an overview of the tissue architecture**. (B) Exemplary cutting lines of laser microdissection. (C) Stroma areas with about 100 square μm were cut out using the laser microdissection and transferred on a ProteinChip array (D). The same procedure was performed with normal connective tissue (not to scale).

From these samples 12 μm cryostat sections were prepared. One section was stained with hematoxylin-eosin (HE) and examined microscopically in order to detect tissue areas of interest for microdissection (see [11]). A corresponding unstained tissue section was mounted on a microscope slide coated with a 1.35 μm membrane (polyethylene naphtalate (PEN) Zeiss/Palm, Bernried, Germany). Tissue areas from normal and tumor stroma (approx. size 300 × 300 μm) containing approximately 100 to 500 cells were cut out and moved by a laser microdissection and pressure catapulting microscope (LMPC; Zeiss/Palm, Bernried, Germany) or a fine needle directly on ProteinChip arrays (Fig. 1). For catapulting, a microplasma is induced under the dissected tissue area. This plasma lifts the piece of tissue to a reaction cup or to a ChipArray fixed by a special mount, each. For regular formed tissue pieces with more than 100 cells we found that it is more secure to attach the dissected area to a fine needle and deposit it elsewhere under microscopically control.

### Applying microdissected tissue onto ProteinChip arrays and mass spectrometric analysis

A Q10 ProteinChip array (strong anion exchanger; BioRad) was activated (see [11]) and wetted with 0.5 μl lysis buffer (100 mM Na-phosphate (pH 7.5), 5 mM EDTA, 2 mM MgCl_2_, 3 mM 2-β-mercaptoethanol, 0.1% CHAPS, 500 μM leupeptine, and 0.1 mM PMSF). Under a stereo microscope (Stemi 2000c, Zeiss) the tissue section was placed on the spot of the ProteinChip array. Tissue lysis on spot was performed for 1.5 h at 4°C in a humidity chamber. After lysis and incubation the spots were washed three times with 5 μl of a washing/binding buffer (100 mM Tris-buffer, pH 8.5 with 0.02% Triton X-100) and rinsed 2 times with water. 2 × 0.5 μl sinapinic acid (saturated solution in 0.5% TFA/50% acetonitrile) was applied as matrix on the dried spots. The matrix which co-crystallizes with proteins absorbs the laser energy and transfers part of its charge to the proteins. Mass analysis was performed in a ProteinChip Reader (PCS 4000, Ciphergen Biosystems Inc, Fremont, CA) with a manual data collection protocol.

Because cells were microdissected, placed and lysed directly on the spot of the ProteinChip array under control of a stereo microscope, we named this technique 'tissue on chip based mass spectrometry' (toc-MS). Areas of different size and cell number were tested. At least 100 cells were needed for an adequate spectrum. For the analysis of the normal and tumor samples 300 cells were dissected for more robust results. Compared to the SELDI standard procedure the sensitivity is increased at least tenfold and, because no protein lysis and extraction is needed, time of analysis is shorter by half. In contrast to MALDI imaging, which allows to analyse spatial resolved protein spectra over tissue sections and other mass spectrometry techniques, the SELDI characteristic affinity chromatograhic chip surfaces allow a more quantitative analysis of proteins.

### Bioinformatic analysis of mass spectrometry data

**Figure 2:**
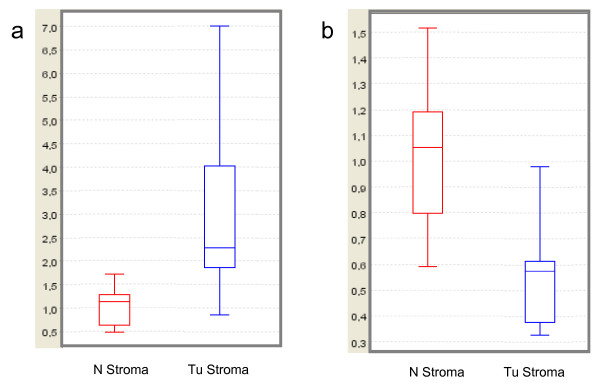
**A: Example of a peak (7.48 kDa) significantly higher expressed in tumor (Tu) stroma compared to normal (N) stroma**. Intensity is plotted on X-Axis. b: Example of a peak (89.04 kDa) significantly lower expressed in tumor (Tu) stroma compared to normal (N) stroma. Intensity is plotted on X-Axis

The resulting protein profiles between 2 kDa to 20 kDa (low range) and 20 kDa to 200 kDa (high range) were subjected to CiphergenExpress™ Client 3.0 software (CE) and a cluster and rule-based data mining algorithm (XLminer 3.0, BioControl Jena GmbH). The CE software was used for the processing of raw spectra and the calculation of *P*-values and cluster plots. In the low range we found 8 peaks with a *P*-value lower 0.05. In the high range 5 peaks with this characteristic could be found. The two most significant proteins for the low and high range are displayed in box plots in Figure 2.

The 7,477 Da peak is significantly higher expressed (*P *= 0.0003) in tumor stroma, while the 80,044 Da peak (*P *= 0.0009) is reduced in tumor stroma. An initial data base search according molecular size offered for the 7,477 Da mass the beta defensin 119 (UniProtKB/Swiss-Prot Q8N690 Chain: 22-84: 7493 Da). Human beta-defensines (HBD) are cationic, antimicrobial peptides produced by epithelial cells and show altered inconsistent expression in cancers [12,13]. Analyses of their expression in tumor stroma are not published yet. For the fibroblast growth factor 23 only a role in phosphate homeostasis and related disorders is known [14]. The protein with a molecular mass of 80,044 Da is equivalent in size to the unphosphorylated ski oncogene (UniProtKB/Swiss-Prot P12755, 80,005 Da) which was discovered as oncogene by its ability to transform chicken embryo fibroblasts upon overexpression. But in newer studies also anti-oncogenic activities are discussed (for review see [15]).

**Figure 3:**
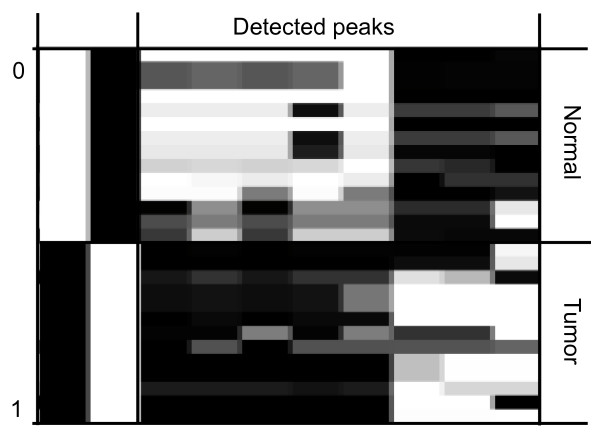
**XLminer heat map including stroma from normal (n = 14) und tumor (n = 13) samples (horizontal rows)**. In columns the relevant features (peaks) are displayed. The analysis resulted in a sensitivity of up to 92.8% and a specificity of 100%.

The subsequent modified fuzzy c-means data analysis algorithm underlying the XLminer software [5] consists of three steps in particular allowing adequate analysis of small sample groups. The clustering step, the rule extraction and rating step, and the rule-base construction step finally result in a heat-map and in values for sensitivity and specificity separating both groups. The analysis of all tumor and normal samples with XLminer resulted in a sensitivity of up to 92.8% and a specificity of 100% (Fig. 3).

In conclusion, we applied toc-MS successfully to analyse a few hundred stromal cells quantitatively and to differentiate between those stromal areas near to tumor and to normal epithelium. An exact identification of these proteins with tryptic digestion and tandem MS is in progress. Ongoing research focuses on down-scaling the procedure to a higher sensitivity.

## Abbreviations

MS: mass spectrometry; toc-MS: tissue on chip based mass spectrometry; MALDI: matrix-assisted laser desorption and ionization; SELDI: surface enhanced desorption and ionization; CAF: cancer associated fibroblasts; TEC: tumor endothelial cells; TAM: tumor associated macrophages; MMP: matrix metalloproteinases; LMPC: laser microdissection and pressure catapulting.

## Competing interests

The authors declare that they have no competing interests.

## Authors' contributions

NE performed SELDI experiments. GE performed tissue microdissection. CM supervised SELDI experiments and did database research. AB performed pathological examination and classification of tissues; critical reading of manuscript. JHC performed conception, single cell work, data collection and writing manuscript. KJ performed conception, interpretation of data and writing manuscript. KF performed conception, biochemical work, interpretation of data and writing manuscript. OGL looked for adequate tumor samples and clinical aspects and writing manuscript. FvE performed conception, design, supervision and writing manuscript. All authors read and approved the final manuscript.
